# Casein kinase-1γ1 and 3 stimulate tumor necrosis factor-induced necroptosis through RIPK3

**DOI:** 10.1038/s41419-019-2146-4

**Published:** 2019-12-04

**Authors:** Song-Yi Lee, Hyunjoo Kim, Cathena Meiling Li, Jaemin Kang, Ayaz Najafov, Muhah Jung, Soosung Kang, Shaomeng Wang, Junying Yuan, Yong-Keun Jung

**Affiliations:** 1 School of Biological Science, Seoul National University, 1 Gwanak-ro, Gwanak-gu, Seoul, 08826 Korea; 2 Department of Cell Biology, Harvard Medical School, 240 Longwood Avenue, Boston, MA 02115-5730 USA; 3 Department of Pharmacy, Ewha Womans University, 52 Ewhayeodae-gil, Seodaemun-gu, Seoul, 03760 Korea; 4 Department of Pharmacy, University of Michigan, 210 Washtenaw Avenue, Ann Arbor, MI 48109-2216 USA

**Keywords:** Necroptosis, Post-translational modifications

## Abstract

Upon necroptosis activation, receptor interacting serine/threonine kinase (RIPK)1 and RIPK3 form a necrosome complex with pseudokinase mixed lineage kinase-like (MLKL). Although protein phosphorylation is a key event for RIPK1 and RIPK3 activation in response to a necroptosis signal, relatively little is known about other factors that might regulate the activity of these kinases or necrosome formation. Through a gain-of-function screen with 546 kinases and 127 phosphatases, we identified casein kinase 1 gamma (CK1γ) as a candidate necroptosis-promoting factor. Here, we show that the decreased activity or amounts of CK1γ1 and CK1γ3, either by treatment with a chemical inhibitor or knockdown in cells, reduced TNFα-induced necroptosis. Conversely, ectopic expression of CK1γ1 or CK1γ3 exacerbated necroptosis, but not apoptosis. Similar to RIPK1 and RIPK3, CK1γ1 was also cleaved at Asp^343^ by caspase-8 during apoptosis. CK1γ1 and CK1γ3 formed a protein complex and were recruited to the necrosome harboring RIPK1, RIPK3 and MLKL. In particular, an autophosphorylated form of CK1γ3 at Ser^344/345^ was detected in the necrosome and was required to mediate the necroptosis. In addition, in vitro assays with purified proteins showed that CK1γ phosphorylated RIPK3, affecting its activity, and in vivo assays showed that the CK1γ-specific inhibitor Gi prevented abrupt death in mice with hypothermia in a model of TNFα-induced systemic inflammatory response syndrome. Collectively, these data suggest that CK1γ1 and CK1γ3 are required for TNFα-induced necroptosis likely by regulating RIPK3.

## Introduction

Necroptosis is an important necrotic cell death mechanism under apoptosis-deficient conditions^1–4^. Necroptosis is involved in many pathological conditions such as sterile inflammation, neurodegenerative diseases, and abortion of defective embryos during embryonic development^5–8^. Receptor interacting serine/threonine kinase (RIPK)1 and RIPK3 were identified as two decisive serine/threonine kinases mediating necroptosis. Caspase-8 inactivates RIPK1 and RIPK3 by proteolytic cleavage;^9,10^ thus, prevention of this cleavage with either an inhibitor or genetic deletion of caspase-8 allows for RIPK1 and RIPK3 activation, leading to formation of a necroptosis-initiating complex with pseudokinase mixed lineage kinase-like (MLKL), which is referred to as the necrosome^11^.

The activation of RIPK1 and RIPK3 upon necroptosis is marked by their phosphorylation. Multiple serine/threonine residues in RIPK1 and RIPK3 can be phosphorylated, which may positively or negatively regulate their activities^12–14^. Among them, the phosphorylation of Ser^166^ is a biomarker of RIPK1 activation^12,15^ and the phosphorylation of RIPK3 Ser^227^ is required for the recruitment and activation of MLKL^16^. Recently, a few kinases and phosphatases other than RIPK1 and RIPK3 have also been reported to regulate the phosphorylation of RIPKs. For example, mitogen-activated protein kinase-activated protein kinase 2 (MK2) can directly phosphorylate RIPK1 and inhibit its activity^17,18^. Transforming growth factor β-activated kinase 1 (TAK1) was also shown to phosphorylate RIPK1 along with IKKε to prevent tumor necrosis factor (TNF)-induced necroptosis^19^ or to dictate the multiple cell death pathways: RIPK1-independent apoptosis or RIPK1/RIPK3-dependent necroptosis^20^. Besides IKKε, IKKα/IKKβ is also able to phosphorylate RIPK1 in order to block RIPK1-dependent cell death^21^. In addition, protein phosphatase 1B (PPM1B) acts as a negative regulator to suppress necroptosis by dephosphorylating RIPK3^22^.

Casein kinase (CK)1γ is an isoform of the CK1 family of serine/threonine kinases. Seven CK1 isoforms (α, β, γ1, γ2, γ3, δ, and ε) and several splice variants have been found in vertebrates^23^. All of these isoforms share a highly conserved N-terminal kinase domain and a C-terminal domain that determines the substrate specificity and kinase activity. Despite the identification of many proteins that are phosphorylated by CK1 family members and their participation in various cellular processes such as Wnt signaling, cell cycle control, DNA repair, and apoptosis^24^, little is known about the role of CK1γs in necroptosis.

To discover new factor(s) involved in the phosphorylation-mediated progression of necroptosis, in this study, we carried out a gain-of-function (GOF) screen using a cDNA expression library encoding kinases and phosphatases. Here, we show that CK1γ1 and CK1γ3 are recruited to the necrosome in response to necroptosis and exacerbate TNFα-induced necroptosis both in vitro and in vivo.

## Results

### Functional screen identified CK1γ as a mediator of TSI-induced necroptosis

**Fig. 1:**
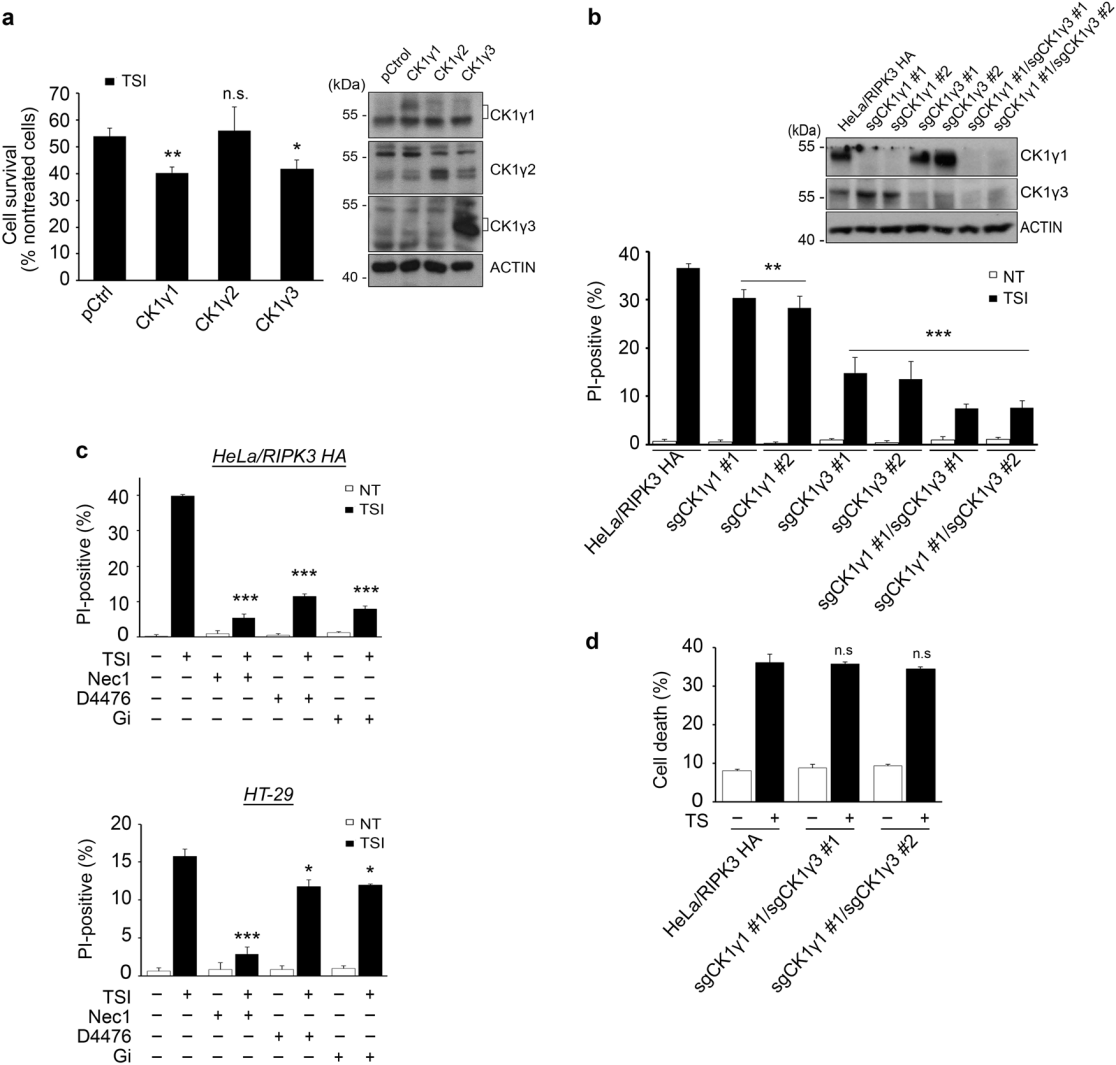
CK1γ is required for TSI- induced necroptosis. Functional isolation of CK1γ as a regulator promoting TSI-induced necroptosis. **a** Ectopic expression of CK1γ1 or CK1γ3 enhances necroptosis. HeLa cells stably expressing RIPK3-HA were transfected with pcDNA3 (control), CK1γ1, CK1γ2 or CK1γ3 for 24 h, and then treated with 20 ng/mL TNFα (T), 100 nM Smac-mimetic SM-164 (S) and 20 μM IDN-6556 (I) for 6 h. The cell viability was determined by CellTiter-Glo (left). The expression levels of CK1γ1, CK1γ2, and CK1γ3 were detected by western blotting (right). Bars represent the mean ± SEM from at least four independent experiments. An unpaired t-test was done in SPSS Statistics. **b** Knockdown of CK1γ1 and CK1γ3 expression inhibits necroptosis. HeLa/RIPK3-HA/CK1γ knockout (sgCK1γ1, CK1γ3 or both CK1γ1 and CK1γ3) cells were treated with 10 ng/mL TNFα, 100 nM SM-164 and 10 μM IDN-6556 for 3.5 h (lower). The expression levels of CK1γ1 and CK1γ3 were detected by western blotting (upper). Bars represent the mean ± SEM from at least three independent experiments. **c** CK1γ inhibitor blocks necroptosis. HeLa/RIPK3-HA (upper) or HT-29 (lower) cells were treated with 10 ng/mL TNFα (upper, HeLa/RIPK3-HA) or 20 ng/mL TNFα (below, HT-29), 100 nM SM-164 and 10 μM IDN-6556 for 3.5 h or 6 h, respectively, in the presence or absence of 10 μM Nec1, D4476 or Gi. Cell death rates were determined by counting propidium iodide (PI)-positive cells. Bars represent the mean ± SEM from at least four independent experiments. **d** Downregulation of CK1γ has no effect on TS-induced apoptosis. HeLa/RIPK3 HA/ CK1γ DKO cells were treated with 20 ng/mL TNFα and 100 nM SM-164 for 7 h. Cell viability was determined by trypan blue exclusion assay. Bars represent the mean ± SEM from at least three independent experiments. *p < 0.05, **p < 0.01, ***p < 0.001, n.s.; not significant.

To identify factor(s) involved in TNFα-induced necroptosis, we took advantage of a cell-based functional assay with a cDNA expression library. Because phosphorylation is known to play an important role in the activation of RIPK1 and RIPK3, we analyzed a collection of expression vectors for full-length cDNAs encoding 546 kinases and 127 phosphatases using a GOF assay. HeLa cells stably expressing RIPK3-HA (HeLa/RIPK3-HA) were generated and transfected with an expression vector for each cDNA individually and then exposed to TNFα (T), Smac-mimetic (S) and IDN-6556 (I) to induce necroptosis. Through primary and secondary screen, we found putative positive clones that affected the rate of necroptotic cell death (Table S1). Among the clones, CK1γ1 was identified as the most efficient promoter of necroptotic cell death. Because CK1γ has three isoforms (CK1γ1, CK1 γ2, and CK1γ3), we tested the effects of each isoform on necroptosis. When ectopically overexpressed in HeLa/RIPK3-HA cells, both CK1γ1 and CK1γ3 significantly enhanced the rate of cell death, while CK1γ2 had no such effect (Fig. 1a). In addition, CK1γ1 and CK1γ3, but not CK1γ2, accumulated in necroptotic cells (Supplementary Fig. S1). We also found that the increased levels of CK1γ during necroptosis were reduced by the reactive oxygen species (ROS) scavenger N-acetyl-l-cysteine (NAC) (Supplementary Fig. S2).

We next tested the effects of CK1γs knockout on necroptosis. When CK1γ1 was knocked out alone using the CRISPR/Cas9 system in HeLa/RIPK3-HA cells, it had a partial rescue effect on cell death (27% reduction) (Fig. 1b, lower). Interestingly, necroptotic cell death was more efficiently reduced by CK1γ3 knockdown in HeLa/RIPK3-HA cells (63% reduction). Compared to that induced by CK1γ1 or CK1γ3 knockdown alone, cell death was further suppressed by CK1γ1 and CK1γ3 double knockout in HeLa/RIPK3-HA cells (84% reduction). Notably, when CK1γ1 expression was reduced in HeLa/RIPK3-HA cells, we observed a compensatory increase of CK1γ3 expression in the CK1γ1 knockout cells (Fig. 1b, upper).

In addition, we used chemical inhibitors of CK1γ to confirm its roles in necroptosis. D4476 is a cell-permeable inhibitor that is widely used to block the kinase activity of the CK1 family^25^, while Gi is a specific of inhibitor of CK1γ isoforms^26^. Compared to necrostatin-1 (Nec1), an inhibitor of RIPK1 kinase activity^12,27^, Gi as well as D4476 exerted a cytoprotective effect against TSI-induced necroptosis in both HeLa/RIPK3-HA and HT-29 cells, although the CK1γ inhibitors were not as efficient at relieving cell death in HT-29 cells (Fig. 1c). We also confirmed that for a longer period, D4476 and Gi were still competent in inhibiting necroptosis (Supplementary Fig. S3). Next, to examine if CK1γ could also affect other types of cell death, we induced apoptosis in HeLa cells with TNFα plus Smac-mimetic, etoposide, or tunicamycin. In contrast to its effect on necroptosis, CK1γ1 had no effect on apoptosis induced under these conditions (Supplementary Fig. S4). Additionally, we verified that the downregulation or inhibition of CK1γ had no effect on TS-induced apoptosis (Fig. 1d and Supplementary Fig. S5). Collectively, these results suggest that both CK1γ1 and CK1γ3 are critical in TSI-induced necroptosis, but not apoptosis.

### CK1γ1 is cleaved by caspase-8 in vitro and in apoptotic cells

**Fig. 2:**
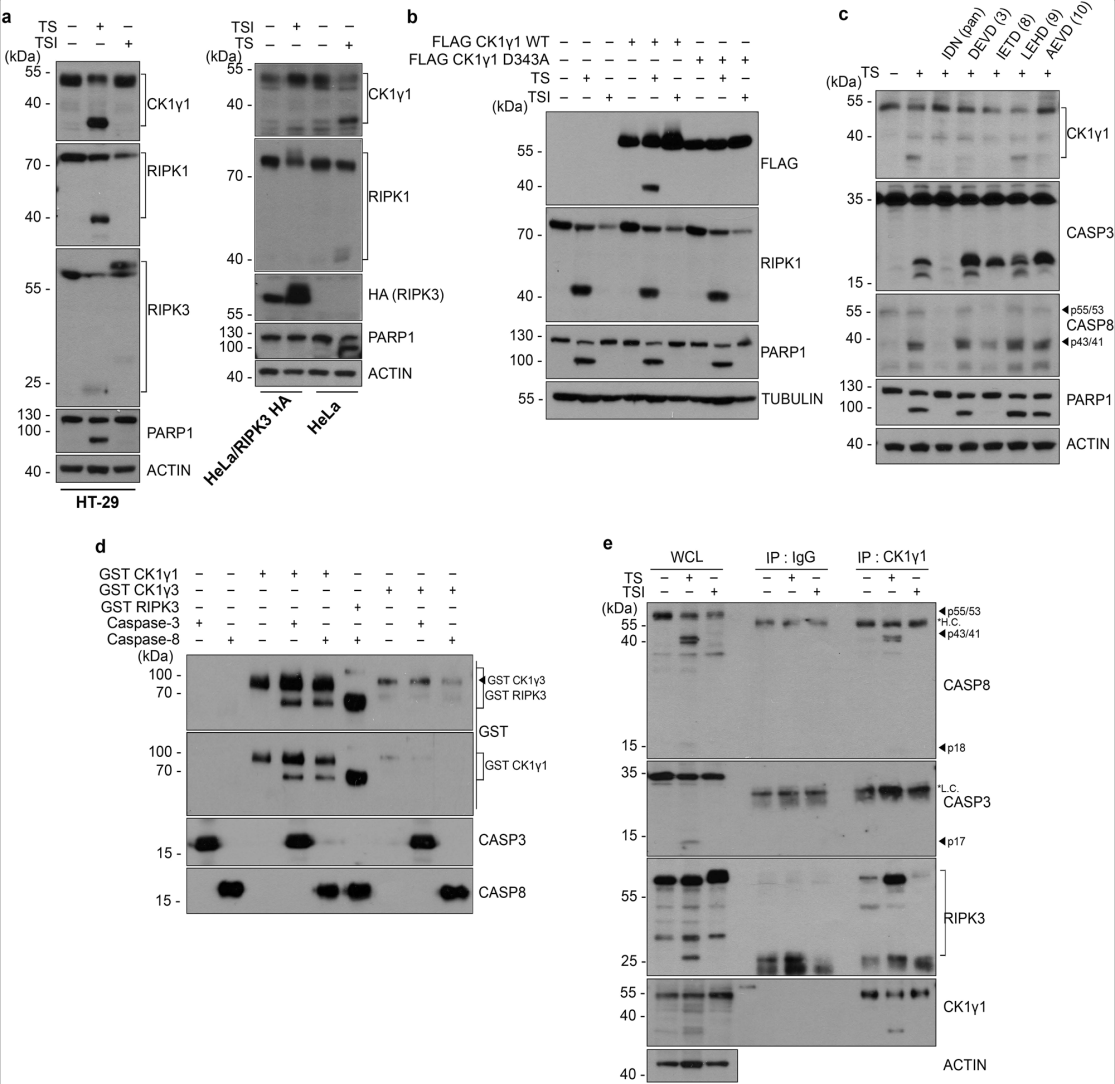
CK1γ1 is cleaved by caspase-8 in vitro and in apoptotic cells. **a** CK1γ1 is cleaved during apoptosis. HT-29 (left), HeLa and HeLa/RIPK3-HA (right) cells were treated with 20 ng/mL TNFα and 100 nM SM-164 to induce apoptosis or with 10 ng/mL TNFα, 100 nM SM-164 and 10 μM IDN-6556 to induce necroptosis. Cell extracts were analyzed by western blotting. **b** CK1γ1 is cleaved at Asp^343^ by caspase. HeLa cells were transfected with FLAG-CK1γ1 WT or non-cleavable CK1γ1 D343A mutant for 24 h, exposed to TS in the presence or absence of 10 μM IDN-6556 for 6 h and cell extracts were examined by western blotting. **c** CK1γ1 cleavage is inhibited by caspase-8 or 3 inhibitor. HeLa cells were treated with TS in the presence of the indicated caspase inhibitor (10 μM). **d** CK1γ1 is cleaved by caspase-8 or caspase-3 in vitro. Recombinant GST-RIPK3, GST-CK1γ1 and GST-CK1γ3 proteins were incubated for 2 h with purified caspase-3 or 8 protein for in vitro cleavage assay. **e** CK1γ1 binds to active caspase-8, not to caspase-3, in apoptotic cells. HeLa/RIPK3-HA cells were treated with TS for 6 h or TSI for 3 h, and then analyzed by immunoprecipitation (IP) assay using anti-CK1γ1 antibody.

RIPK1 and RIPK3 are known as targets of caspase-8 and are inactivated by cleavage during apoptosis. Similar to the cleavage of RIPK1 and RIPK3, we observed the cleavage of CK1γ1 during apoptosis. The cleavage product of CK1γ1 with a molecular weight of 37 kDa appeared in TS-induced apoptotic HT-29 cells, but not in TSI-induced necrotic cells (Fig. 2a, left). PARP, a well-known caspase substrate, was also found to be cleaved in the apoptotic cells. Likewise, the cleavage of CK1γ1 was also observed in HeLa cells undergoing TS-induced apoptosis but not in HeLa/RIPK3-HA cells undergoing necroptosis (Fig. 2a, right). These cleavages triggered by TS were no longer observed in the presence of the pan-caspase inhibitor IDN-6556 (Fig. 2a, b). Based on the size of the caspase cleavage product and with the help of the program “Cascleave” (http://sunflower.kuicr.kyoto-u.ac.jp/sjn/Cascleave/webserver.html) to predict the caspase cleavage motif, we chose Asp^343^ as a probable cleavage site. Subsequent mutagenesis analysis revealed that the CK1γ1 D343A mutant, in which Asp^343^ is replaced with Ala, was resistant to the cleavage during apoptosis (Fig. 2b), showing that CK1γ1 is cleaved by caspase during apoptosis. Further, we examined the effect of CK1γ1 wile-type (WT) and D343A expression on cell death in HeLa/RIPK3-HA control and CK1γ1 knockout cells. First of all, we should notice that FLAG-tagged CK1γ1 WT did no longer increase necroptotic cell death in HeLa/RIPK3-HA cells (Supplementary Fig. S6). Note that in Fig. 1a, untagged CK1γ1 WT isolated from the expression library was used. Both FLAG-tagged CK1γ1 WT and CK1γ1 D343A mutant equally increased necroptotic cell death rate in two CK1γ1 knockout cell lines. It is probably because of IDN-6556 that was used to induce necroptosis, thus CK1γ1 WT could avoid a chance to be cleaved by a caspase.

To identify a caspase involved in the cleavage of CK1γ1, we examined the effects of caspase inhibitors on this cleavage. Among them, z-IETD-fmk, an inhibitor of caspase-8, along with IDN-6556, were most effective to prevent the cleavage of CK1γ1, while z-DEVD-fmk, an inhibitor of caspase-3, and z-AEVD-fmk, an inhibitor of caspase-10, showed less effectiveness (Fig. 2c). Although z-DEVD-fmk did not completely block PARP1 cleavage, it nevertheless affected the cleavage of CK1γ1 to some degree. Therefore, we further tested if the candidate caspases could cleave CK1γ1 in a cell-free system. For this experiment, recombinant RIPK3 protein was used as a control target of caspase-8. Under this condition, both purified caspase-3 and caspase-8 proteins cleaved CK1γ1 to produce the GST-CK1γ1 cleavage product in vitro (Fig. 2d). By contrast, the immunoprecipitation assay revealed that only caspase-8 interacted with CK1γ1 in apoptotic cells (Fig. 2e), indicating that CK1γ1 is cleaved by caspase-8 during apoptosis. In addition, we checked whether CK1γ3 was cleavable by caspase. Unlike CK1γ1, in vitro cleavage and apoptotic assays revealed that CK1γ3 was not cleaved by either caspase-3 or caspase-8 (Fig. 2d). Collectively, these results suggest that CK1γ1 and CK1γ3 are regulated differently, at least by caspase-8, although they act together in mediating necroptosis.

### CK1γ is a component of the necrosome harboring active RIPK1, RIPK3, and MLKL

**Fig. 3:**
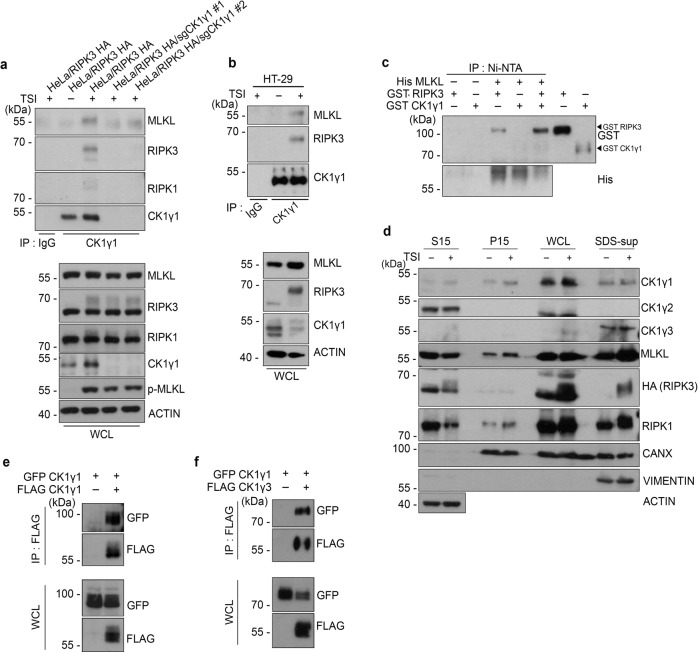
Upon necroptosis, CK1γ interacts with necrosome and is found in a subcellular fraction harboring active RIPK1, RIPK3 and MLKL. **a**, **b** CK1γ1 interacts with RIPK1, RIPK3 and MLKL in necroptotic cells. HeLa/RIPK3-HA or HeLa/RIPK3-HA/CK1γ1 knockout (**a**) or HT-29 (**b**) cells were treated with 20 ng/mL TNFα, 100 nM SM-164 and 10 μM IDN-6556 for 3 h (**a**) or 6 h (**b**), respectively, and analyzed by immunoprecipitation (IP) assay with anti-CK1γ1 antibody. The immunoprecipitants (upper) and whole cell lysates (WCL) (lower) were analyzed by western blotting. **c** In vitro assay showing the binding of CK1γ to MLKL and RIPK3. Recombinant His-MLKL, GST-RIPK3 and GST-CK1γ1 proteins were incubated overnight at 4 °C as indicated and analyzed by immunoprecipitation (IP) assay using Ni-NTA beads. **d** CK1γ1 and CK1γ3 are detected in a highly insoluble fraction harboring RIPK1, RIPK3 and MLKL. HeLa/RIPK3-HA cells were treated with TSI for 3 h and fractionated by centrifugations at 15,000 rpm into supernatates (S15) and pellet (P15) or WCL and SDS-sup fractions, which were obtained from the pellets of WCL and dissolved by 1% SDS first, then further diluted with lysis buffer to 0.1% SDS. Each cellular fraction was analyzed by western blotting. **e**, **f** GFP-CK1γ1 makes up homocomplex with FLAG-CK1γ1 and heterocomplex with FLAG-CK1γ3. HEK 293T cells were cotransfected with GFP-CK1γ1 and FLAG-CK1γ1 (**e**) or GFP-CK1γ1 and FLAG-CK1γ3 (**f**), and subjected to immunoprecipitation (IP) assay with anti-FLAG beads. Whole cell lysates (WCL) and the immunoprecipitates were analyzed by western blotting.

Next, we examined if CK1γ was a component of the necrosome complex. To explore this possibility, an immunoprecipitation assay was first performed using anti-CK1γ1 antibody because CK1γ3 was not detected in the 1% Triton X-100-soluble fraction (WCL in Fig. 3d). Upon treatment with TSI, CK1γ1 was recruited into the immunocomplex containing RIPK1, RIPK3, and MLKL in HeLa/RIPK3-HA cells (Fig. 3a). Similarly, we found the protein complexes harboring active CK1γ1, RIPK3 and MLKL in HT-29 cells actively undergoing TSI-induced necroptosis (Fig. 3b). To verify their interactions, we conducted an in vitro binding assay using purified proteins. When His-MLKL protein was pulled-down using Ni-NTA beads, only small amounts of CK1γ1 were detected in the MLKL complex regardless of whether or not it was incubated with RIPK3. Of note, the binding of RIPK3 to MLKL was enhanced in the presence of CK1γ1 (Fig. 3c). However, compared to that of MLKL and RIPK3, the interaction between MLKL and CK1γ1 was relatively weak. CK1γ3 also showed similar patterns with CK1γ1 (Supplementary Fig. S7). Together, these results imply that CK1γ1 or CK1γ3 might preferentially bind to RIPK3, rather than MLKL, which facilitates formation of the MLKL and RIPK3 complex.

Activation of RIPK1 and RIPK3 by necroptotic stimuli results in the formation of an amyloid-like structure, which is found as a highly insoluble fraction^28,29^. Thus, using a cellular fractionation assay, we examined whether CK1γ could bind to the necrosome in the insoluble fraction. As previously reported, RIPK1, RIPK3 and MLKL moved toward the more insoluble fraction (P15 or SDS-sup) under stimulation with necroptotic stimuli (Fig. 3d). By contrast, the subcellular localizations of CK1γs were not changed upon necroptosis. CK1γ1 was located mainly in the 1% Triton X-100-soluble fraction (WCL) with some detected in the P15 and the sodium dodecyl sulfate-supernatant (SDS-sup) fractions, whereas CK1γ3 was detected only in the SDS-sup fraction where active necrosomes were localized most abundantly. Unlike CK1γ1 and CK1γ3, CK1γ2 was exclusively found in the S15 fraction, whose components were the most soluble, overall. These results suggest that CK1γ1 and CK1γ3 might function in necroptosis after activated RIPK1, RIPK3 and MLKL move to the location of CK1γ. In addition, homotypic and heterotypic interactions between CK1γ1 and CK1γ3 were observed when they were ectopically expressed in HEK 293T cells (Fig. 3e, f).

### CK1γ is autophosphorylated and can phosphorylate RIPK3 to activate it in vitro

**Fig. 4:**
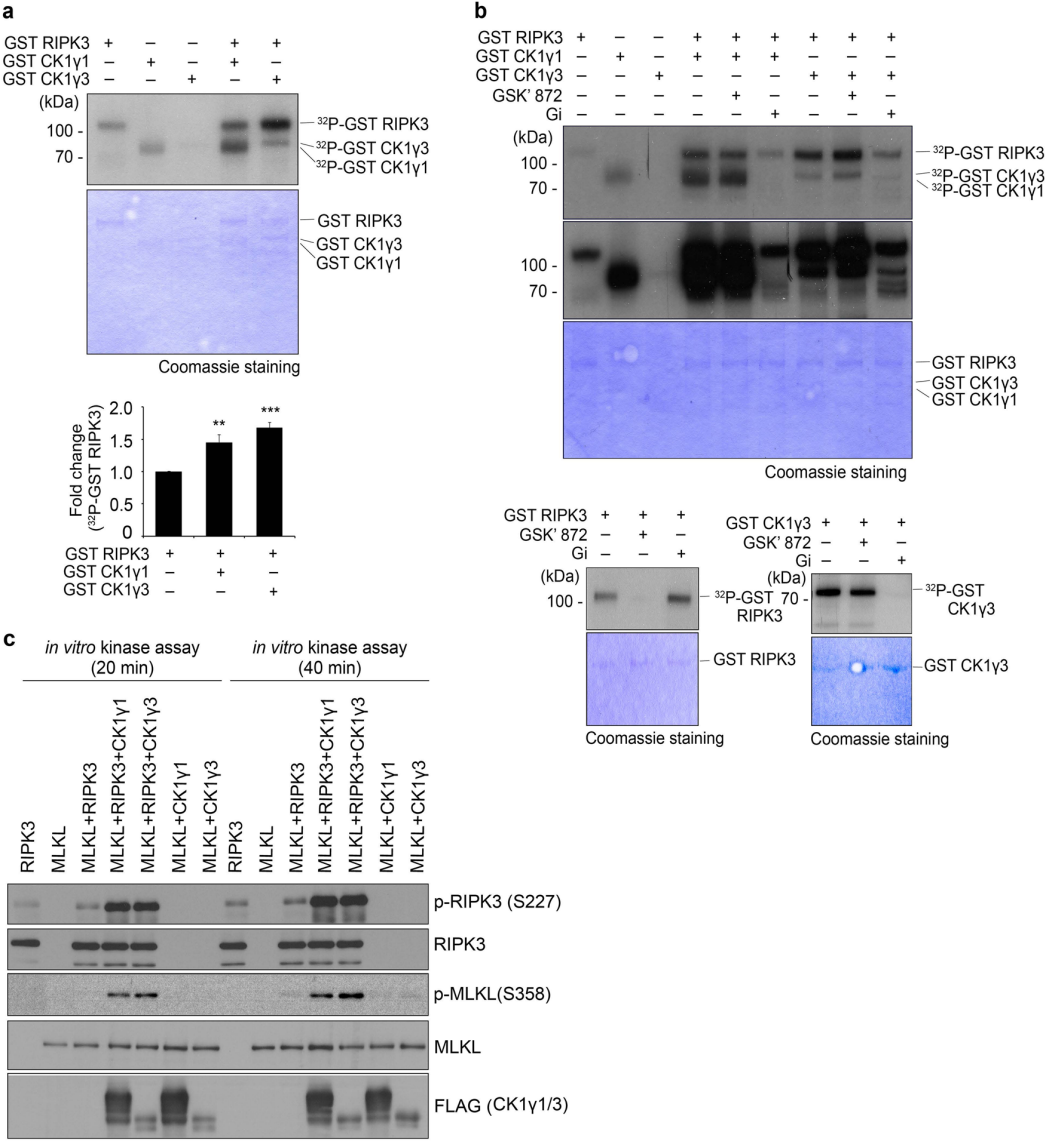
CK1γ is auto-phosphorylated and phosphorylates RIPK3 to activate it in vitro. **a** In vitro kinase assay showing CK1γ autophosphorylation and increase of RIPK3 phosphorylation by CK1γ. Recombinant GST-RIPK3, GST-CK1γ1 and GST-CK1γ3 proteins were incubated as indicated for 2 h in a kinase assay with 10 mCi [^32^p] ATP. The reaction mixtures were separated by SDS-PAGE and transferred to nitrocellulose membrane, followed by autoradiography (top). Purified proteins used were stained by Coomassie blue (middle). Bars represent the mean ± SEM from at least six independent experiments (bottom). **p < 0.01, ***p < 0.001. **b** CK1γ inhibitor Gi blocks RIPK3 phosphorylation by CK1γ (upper and lower right) but not RIPK3 auto-phosphorylation (lower left). The same kinase assays were performed in the absence or presence of 20 μM GSK'872 or Gi. **c** CK1γ1 and CK1γ3 proteins enhance RIPK3 activity in vitro. Purified proteins were incubated for in vitro assays as indicated. The reaction products were then analyzed by western blotting. RIPK3 activity was determined using anti-RIPK3 p-S227 and anti-MLKL p-S358 antibodies, following its phosphorylation by CK1γ1 and CK1γ3.

Because both RIPK3 and CK1γ are protein kinases, we examined if one of the kinases phosphorylates the other in vitro. In preliminary experiments, we found that CK1γ1 or CK1γ3, as well as RIPK3, are autophosphorylated (Fig. 4a, upper). More importantly, compared to RIPK3 alone (lane 1), the phosphorylation of RIPK3 was enhanced by 1.5- and 1.7-fold when co-incubated with CK1γ1 (lane 4) or CK1γ3 (lane 5), respectively (Fig. 4a, lower). In addition, the phosphorylation of CK1γ1 and CK1γ3 was also somewhat enhanced when incubated with RIPK3. We then decided to identify the kinases responsible for these phosphorylations using chemical inhibitors against RIPK3 (GSK'872) or CK1γ (Gi) (Fig. 4b). Gi and GSK'872 did not affect the phosphorylation of RIPK3 and CK1γ, respectively, in vitro (Fig. 4b, lower). We confirmed that Gi did not show cross reactivity toward the phosphorylation of FLAG-RIPK1 and FLAG-RIPK3 in the transfected cells (Supplementary Fig. S8). In addition, Gi had no effect on MLKL-induced cell death (Supplementary Fig. S9). When the reaction mixtures comprising RIPK3 and CK1γ1 or CK1γ3 were incubated with GSK'872 or Gi, only Gi reduced the phosphorylation of RIPK3 in vitro (lanes 6 and 9 in Fig. 4b) with no such effect observed for GSK'872 (lanes 5 and 8). Therefore, we conclude that CK1γ phosphorylates RIPK3.

However, it remained unclear whether or not the enhanced phosphorylation of RIPK3 by CK1γ results in the activation of RIPK3. Once RIPK3 is activated in necroptotic cells, it phosphorylates MLKL, which subsequently forms oligomers that create pores in the plasma membrane^30^. Unfortunately, in the same kinase assay, it was hard to detect the phosphorylation of MLKL when incubated with RIPK3. Alternatively, we checked the activation of RIPK3 and MLKL using phospho-S227 RIPK3 and phospho-S358 MLKL antibodies, respectively and found CK1γ increased the phosphorylation of MLKL by RIPK3 in vitro (Fig. 4c). Additionally, in non-reducing SDS-polyacrylamide gel electrophoresis (PAGE), treatment with Gi appeared to impede the oligomerization of MLKL in necroptotic cells (Supplementary Fig. S10). Nec1 also blocked the formation of MLKL oligomers in the same assay. Furthermore, we tried to check the activation of RIPK3 by examining the slow-migrating RIPK3, which is thought to be phosphorylated form of RIPK3, on SDS-protein gel, but it was hard to see differences in the migration of RIP3K between WT and CK1γ knockout HeLa/RIPK3 HA cells (Supplementary Fig. S11). Nevertheless, we observed that the interaction of the activated RIPK3 with MLKL was apparently reduced in CK1γ1/CK1γ3 double knockout cell compared to that in WT control cells. As a result, the amounts of phosphorylated MLKL were decreased in CK1γ1/CK1γ3 double knockout cells. Together, these results imply that CK1γ stimulates the activation of MLKL during necroptosis most likely through RIPK3.

### CK1γ3 is autophosphorylated at Ser^344/345^ to exert its activity during necroptosis

**Fig. 5:**
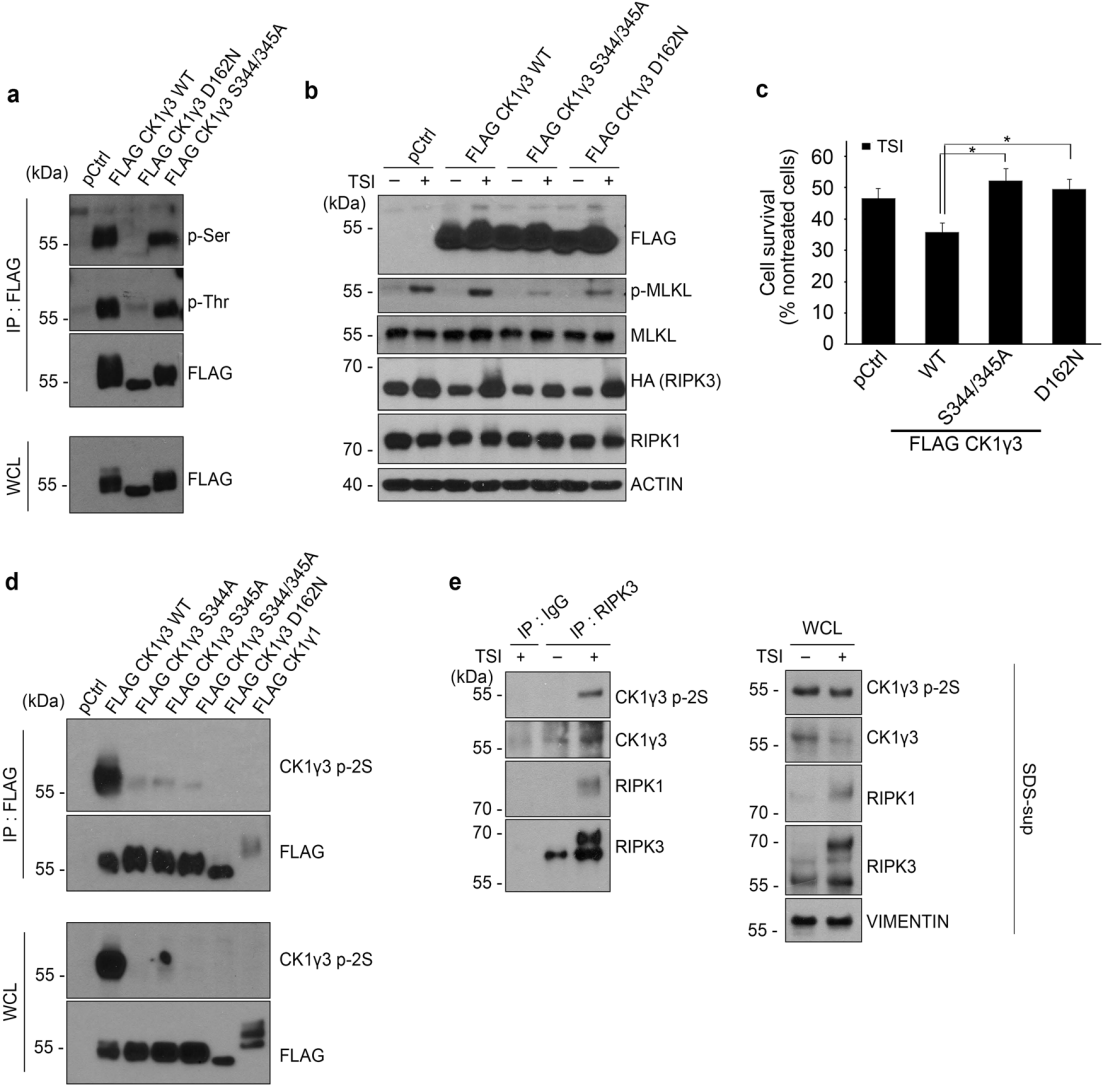
CK1γ3 autophosphorylation occurs at Ser^344/345^ and is important for necroptosis. **a** Mutations at Ser^344/345^ impedes CK1γ3 autophosphorylation. HEK 293T cells were transfected with either FLAG-CK1γ3 WT or its mutant. Cells lysates were analyzed by immunoprecipitation (IP) assay with anti-FLAG beads followed by western blotting using phospho-Ser or phospho-Thr antibody. The immunoprecipitates (upper) and whole cell lysates (lower) were analyzed by western blotting. **b** Overexpressed CK1γ3 undergoes autophosphorylation at Ser^344/345^. HeLa/RIPK3-HA cells were transfected with FLAF-CK1γ3 WT or mutants for 24 h and treated with 10 ng/mL TNFα, 100 nM SM-164 and 10 μM IDN-6556 for 3 h. **c** Overexpression of CK1γ3 Ser344/345Ala mutant reduces necroptosis. HeLa/RIPK3-HA cells were transfected with either CK1γ3 WT or mutants for 24 h, and treated with 20 ng/mL TNFα, 100 nM SM-164 and 20 μM IDN-6556 for 7 h. The cell viability was determined by CellTiter-Glo. Bars represent the mean ± SEM from at least four independent experiments. *p < 0.05. **d** Generation of CK1γ3 p-2S antibody recognizing the phosphorylation at CK1γ3 Ser^344/345^. HEK 293 T cells were transfected with either FLAG-CK1γ3 WT or mutants and analyzed by immunoprecipitation (IP) assay with anti-FLAG beads, followed by western blotting using CK1γ3 p-2S antibody. **e** Phosphorylated form of CK1γ3 Ser^344/345^ is detected in the necrosome of SDS-soluble fraction. HeLa/RIPK3-HA were treated with TSI for 3 h and lyzed in 1% Triton X-100. Undissolved pellets were further lyzed with 1% SDS and finally diluted to 0.1% SDS (SDS-sup fraction) which was used in IP assay.

When CK1γ3 was overexpressed in the cells, we found slow-migrating CK1γ3 proteins on SDS-PAGE gel (Fig. 5a). Given that CK1γ1 and CK1γ3 were found to be autophosphorylated in the in vitro assay (Fig. 4a) and a kinase dead mutant of CK1γ3 (D162N) did not show this delayed migration in SDS-PAGE (Fig. 5a), the slow-migrating CK1γ3 proteins were considered to be phosphorylated forms of CK1γ3. Immunoprecipitation assays using pan phospho-Ser and -Thr antibodies showed that CK1γ3 was phosphorylated at Ser and Thr residues (Fig. 5a). To identify the residues of CK1γ3 phosphorylations, we generated many CK1γ3 mutants in which Ser/Thr residues were replaced with Ala. Among them, introduction of mutations at Ser^344^ and Ser^345^ (CK1γ3 S344/345A) most notably reduced the shift on the protein gel compared to CK1γ3 WT, although the phosphorylation was not completely blocked, as seen for the CK1γ3 D162N mutant (Fig. 5a). We confirmed this phosphorylation at Ser^344/345^ with the help of liquid chromatography tandem-mass spectrometry experiments. Phosphorylated peptides of CK1γ3 at Ser^344/345^ were increased in HeLa/RIPK3-HA cells treated with TSI compared to those of the untreated control (42% and 25%, respectively, Table S2).

We also investigated the role of this phosphorylated CK1γ3 in necroptosis. Compared to CK1γ3 WT, the CK1γ3 S344/345A mutant significantly lost the ability to enhance the TSI-induced phosphorylation of MLKL (Fig. 5b), and therefore reduced the rate of necroptotic cell death (Fig. 5c). We then generated an antibody (CK1γ3 p2S) for detecting the phosphorylated form of CK1γ3 at Ser^344/345^. Western blot analysis showed that the antibody against CK1γ3 p2S well recognized CK1γ3 WT, but not the CK1γ3 S344A, CK1γ3 S345A and CK1γ3 S344/345A mutants as well as the CK1γ3 D162N mutant (Fig. 5d). However, it was hard to find any significant differences in the total levels of phosphorylated CK1γ3 (CK1γ3 p2S) between untreated and TSI-treated cells. There was only a marginal increase in CK1γ3 p2S that simply corresponded to the elevated levels of total CK1γ3 protein in necroptotic cells (Supplementary Fig. S12). Thus, we conducted an immunoprecipitation assay of the necrosome complex as an alternative. Because CK1γ3 was not detected in the WCL faction, as seen in Fig. 3d, we performed the immunoprecipitation assay with anti-CK1γ3 antibody in the SDS-sup fraction. The results revealed that the phosphorylated CK1γ3 at Ser^344/345^ was detected in the RIPK3-containing necrosome of necroptotic cells (Fig. 5e). It thus appears that the phosphorylated form of CK1γ3 at Ser^344/345^ is found in the active necrosome.

### Gi improves survival in a TNFα-induced systemic inflammatory response syndrome mouse model

**Fig. 6:**
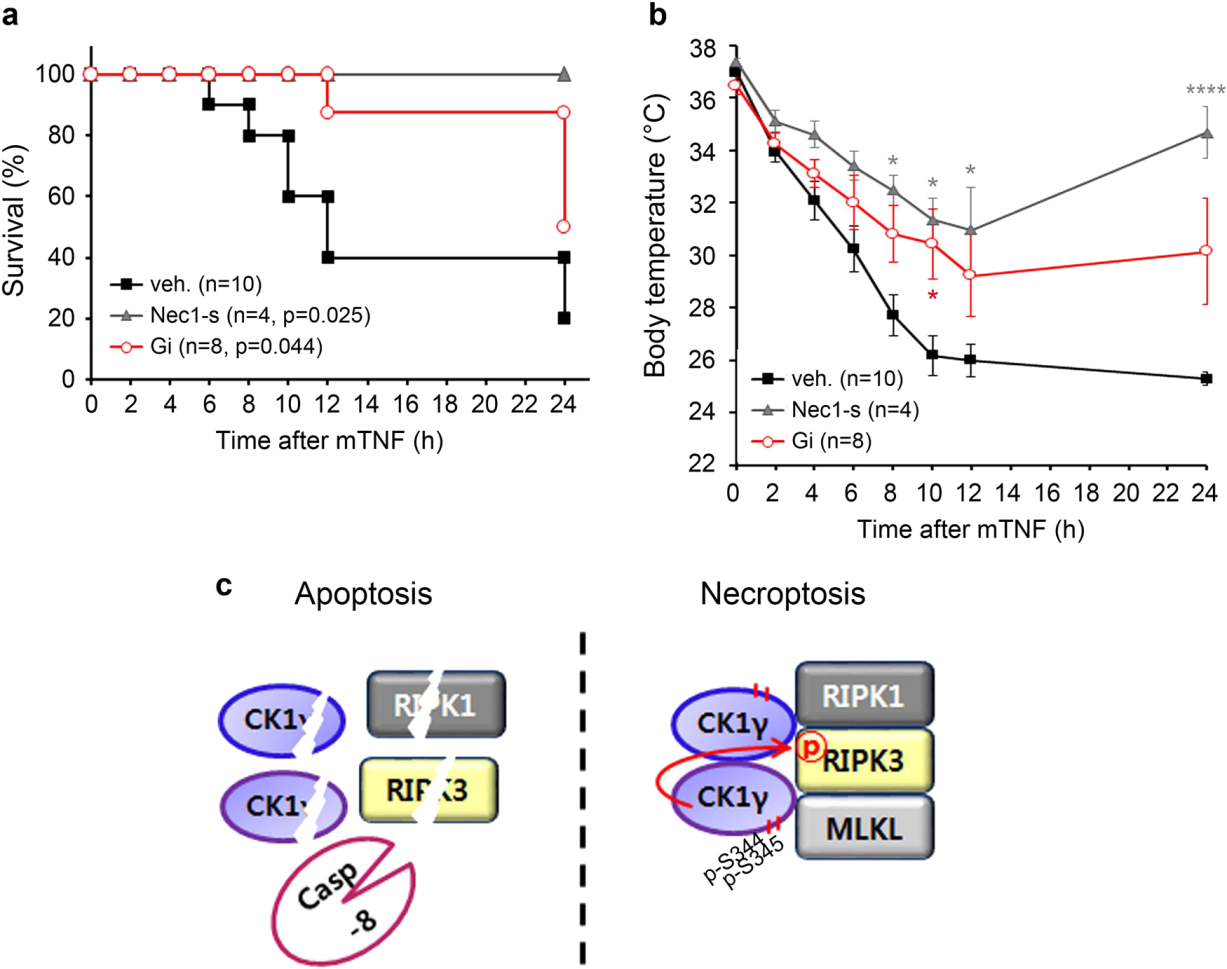
CK1γ inhibitor Gi protects mice against TNFα-induced SIRS. **a**, **b** Tail-vain injection of Gi protects mice form TNFα-induced SIRS. Mice were injected with 3 mg/kg Gi or 7-Cl-O-Nec-1 (Nec1-s) through tail vein for 20 min before challenge with 20 μg mouse (m)TNFα. Mouse survival rate (**a**) and body temperature (**b**) were measured every 2 h using a rectal thermometer probe. Body temperature of dead mice was calculated with 25 °C. Bars represent the mean ± SEM. from at least four independent experiments performed on different days. Kaplan–Meier survival analysis and Gehan-Breslow-Wilcoxon test (**a**) or two-way ANOVA test (**b**) were performed to assess statistical significance. *p < 0.05, ****p < 0.0001. Unless marked, it has no statistical significance. **c** A proposed model for the function of CK1γ in TNFα-induced necroptosis. CK1γ3 is activated by autophosphorylation to mediate necroptosis activation together with CK1γ1 through RIPK3 phosphorylation, while CK1γ1 is cleaved by caspase-8 in apoptosis.

The absence of RIPK1 kinase activity or genetic deletion of RIPK3 has proven to have a protective effect against TNFα-induced hypothermia and to enhance the survival of mice^31,32^. We thus examined the potential in vivo contribution of CK1γ-mediated necroptosis to a TNFα-induced systemic inflammatory response syndrome (SIRS) mouse model. As reported previously^31,32^, injection with mouse TNFα (mTNFα) alone through the tail vein caused the abrupt death of mice with hypothermia (Fig. 6a, b). However, prior injection with 7-Cl-O-Nec-1 (Nec1-s, a more stable analogue of Nec1^12,33^) protected the mice from both death and hypothermia. When the mice were injected with the CK1γ inhibitor Gi prior to mTNFα challenge, the survival rate was improved (Fig. 6a) and hypothermia was also ameliorated, although the effects were slightly weaker than those of Nec1-s (Fig. 6b). These results suggest that the CK1γ inhibitor Gi protects mice with SIRS although less potently than Nec1-s, in line with the results from cell-based assays.

## Material and methods

### Collection of cDNAs and GOF screening

The cDNAs encoding 546 protein kinases and 127 phosphatases were generated from subcloning of the cDNAs into mammalian expression vector, and prepared by a kind gift from other groups and purchase (Origene). HT-29 cells were transfected with GFP and each cDNA for 24 h and then exposed to TSI to induce necroptosis. Based on the morphology of GFP-positive necrotic cells, we first evaluated the death rates of cells expressing kinases or phosphatases. Then, the secondary screening was assessed with similar assay but using with PI to determine the level of necroptosis.

### Cell culture and stable cell lines

HT-29 cells were cultured in McCoy’s 5A medium (Hyclone) and other cells were in Dulbecco’s Modified Eagles Medium (DMEM; Hyclone), supplemented with 10% defined FBS (Gibco) and 100 U/mL penicillin. Cells were maintained in a 37 °C incubator at 5% CO_2_. HeLa cells were transfected with pcDNA3 encoding RIPK3-HA using iN-fect reagents (iNtRON Biotechnology) and selected with G418 (Sigma-Aldrich) to establish the HeLa/RIPK3-HA cell line. CK1γ knockout cells were generated using CRISPR/Cas9 system with LentiCRISPR (pXPR_001) that expresses each gene-targeting sgRNA. HeLa/RIPK-HA cells were transduced with the lentiviral delivery and selected with puromycin (Sigma-Aldrich). For the controls, non-specific sgRNA was transduced into cells with the same procedure.

### Reagents and antibodies

The following chemicals were purchased: human TNFα (Merck Millipore); mouse TNFα (PeproTech); GSK’872, NSA, Nec1-s (Calbiochem); Lipofectamine 2000, TRAIL (Invitrogen); Nec1, etoposide, tunicamycin, (Sigma-Aldrich). Smac mimetic SM-164 was synthesized by Dr. Shaomeng Wang (University of Michigan). Gi was synthesized as described in Hua Z et al.^26^. The antibodies used for the western blot analysis are: anti-RIPK3 (E1Z1D), anti- RIPK1 (p-Ser^166^), anti- RIPK3 (p-Ser^227^), anti-caspase-8, anti-His antibodies (Cell Signaling); anti-CK1γ2, anti-MLKL, anti-caspase-3, anti-vimentin antibodies (Genetex); anti- PARP-1, anti-GST, anti-actin, anti-tubulin, anti-calnexin antibodies (Santa Cruz Biotechnology); anti-FLAG, anti-phospho-serine, anti-phospho-threonine antibodies (Sigma-Aldrich); anti-CK1γ1, anti-phospho-MLKL (p-Ser^358^) antibodies (Abcam); anti-RIPK1 antibody (BD Biosciences) and anti-CK1γ3 (Thermo Fisher Scientific). For the in vitro experiment, GST-RIPK3, GST-CK1γ1 (Sigma-Aldrich), GST-CK1γ3 (Abcam) and His-MLKL (Aviva Systems Biology) proteins were utilized.

### Generation of CK1γ3 phospho-Ser344/345 polyclonal antibody

A phospho-peptide encoding the amino acid residues 339–350 of human CK1γ3 was synthesized and submitted for antibody generation at Abfrontier. Briefly, two rabbits were immunized with the KLH-conjugated antigen twice with 4-week intervals. A week after each injection, the immune serum was collected and tested for immune reactivity by ELISA. Pre-immune serum at week 0 served as negative control. The final sera were affinity purified against the immunogenic peptide and guaranteed for more than 1.5 O.D. value at 1:1,000 in ELISA titer.

### Cell death assay

Cell viability was examined by staining nuclear chromatin with 1 μg/ml propidium iodide (PI; Sigma-Aldrich) after necroptosis induction under a fluorescence microscope (Olympus) or by estimating the ATP level from live cells between NT and TSI-treated groups using CellTiter-Glo (Promega). Apoptotic cell death was determined by trypan blue exclusion assays.

### Immunoprecipitation and subcellular fractionation

Immunoprecipitation and subcellular fractionation were performed following the method previously described^16,29^ with minor modifications. Briefly, to gain S15 and P15 fractions, the harvested cells were resuspended with buffer A (20 mM Hepes pH 7.4, 40 mM KCl, 1.5 mM MgCl_2_, 1 mM EDTA, 1 mM EGTA, 0.1 mM PMSF and 250 mM sucrose) on ice and homogenized with a 22-G needle. After centrifugation at 1000 × *g* for 10 min, the supernatant was again centrifuged at 15,000 × *g* for 10 min. The resulting supernatant was collected as S15 and the pellet was lyzed with lysis buffer (50 mM Tris pH 8.0, 137 mM NaCl, 1 mM EDTA, 1% Triton X-100, and 10% glycerol) and centrifugated to get the supernatant (P15). This P15 fraction was also used for immunoprecipitation assay with anti-CK1γ1 antibodies. The other half of the cells were lyzed with lysis buffer first, and the supernatant was saved as the whole cell extract (WCL). The remaining pellet was resuspended with buffer S (20 mM Tris pH 7.4, 150 mM NaCl, and 1% SDS) and homogenized with a 22-G needle. After centrifugation, the supernatant was saved as SDS-sup.

### Protein purification

In vitro kinase assays were performed, as previously described^34^, with some modifications. pCMV3-N-Flag-CK1γ1 and pCMV3-N-Flag-CK1γ3, or pcDNA3.1-hMLKL-Flag plasmids were transfected into HEK293T cells (per 15 cm dish: 20 µg plasmid + 55 µl PEI + 1 ml OptiMEM, incubate for 15-20 min at 25 °C). Cells were lyzed 48 h later in 0.75 ml of NP-40 lysis buffer (NLB) (25 mM HEPES (pH 7.5), 0.2% NP-40, 120 mM NaCl, 0.27 M sucrose, 2 mM EDTA, 2 mM EGTA, 50 mM NaF, 10 mM beta-glycerophosphate, 5 mM sodium pyrophosphate, 5 mM sodium orthovanadate (added fresh), 0.1% BME (added fresh), 1 mM PMSF (added fresh), 2X Complete protease inhibitor cocktail (Roche, added fresh). After centrifugation at 16,000x g, 15 min, 4 °C, lysates were incubated with anti-Flag-agarose beads for 4 h on a rotating wheel at 4 °C. The beads were washed twice with NLB containing phosphatase inhibitors (each wash for 5 min on a rotating wheel at 4 °C) and twice with a wash buffer containing 1% Triton X-100, 250 mM NaCl, 25 mM Hepes pH 7.4. Flag-tagged proteins were eluted with 0.2 mg/ml Flag peptide for 2 h at 4 °C, on a wheel. RIPK3 was purified from Rosetta™(DE3)pLysS (EMD Millipore) E. coli cells using the plasmid pGEX-4T-1-RIPK3 (http://www.addgene.org/78827/). GST-hRIPK3 was purified as above following *E. coli* lysis by sonication. Elution was made using 40 mM reduced glutathione in PBS.

### In vitro binding assay

Recombinant proteins were incubated in cold phosphate buffered saline (PBS) with 1 mM DTT and 0.2 mM PMSF (Sigma-Aldridch) overnight at 4 °C and analyzed by immunoprecipitation (IP) assay using Ni-NTA beads (GE Healthcare), followed by western blotting.

### In vitro kinase assay

Recombinant proteins were incubated in the kinase buffer (25 mM MOPS pH 7.2, 12.5 mM glycerol-2-phosphate, 25 mM MgC1_2_, 5 mM EGTA, and 2 mM EDTA; 0.25 nM DTT was added just prior to use) for 2 h with 10 mCi [^32^p] ATP (PerkinElmer). The reaction mixtures were separated by SDS-PAGE and transferred to nitrocellulose membrane after the loaded proteins were verified by Coomassie blue staining. Phosphorylations were identified by autoradiography analysis. For in vitro kinase assays using phospho-antibodies, it was performed as described with some modifications^35^. Kinase reaction buffer (25 mM Hepes pH 7.4, 20 mM MgSO4, 2X Thermo’s EDTA-free protease inhibitor cocktail, 10 mM beta-glycerophosphate, 2 mM NaF, 0.1 mM CaCl2, 0.1% BME), purified proteins and ATP (300 µM final concentration) were mixed on ice and the reaction was terminated following 20 min and 40 min shaking at 1200 rpm, at 37 °C, by addition of 5X SDS-PAGE sample buffer and heating at 95 °C for 5 min.

### Statistical analysis

Results are expressed as mean ± S.E.M. and differences were assessed using one-way analysis of variance (ANOVA) with Tukey-adjusted post hoc tests for multiple comparisons unless otherwise stated. All analyses were performed using SPSS Statistics ver.23 software.

## Discussion

The last decade has witnessed great advances in the understanding of necroptosis, a special type of necrosis, mainly due to identification of the RIPK family and its downstream factor MLKL. However, considering the complexity of apoptosis, there is no doubt that other signals and factors besides these molecules are also involved in necroptosis; thus, extensive research effort has been focused on identifying new factors and the detailed mechanism of necroptosis. Toward this end, we used a GOF screening approach with a cDNA expression library. Compared to loss-of-function (LOF) screening using a small interfering RNA library, GOF screening has an advantage of being able to isolate novel factors in such a condition that the signal used for their activation is not operating. In addition, given that CK1γ is a long-lived protein, it is hard to define its function through transient knockdown experiments required for LOF screening. Moreover, at least CK1γ3 is known to functionally complement CK1γ1, as shown in experiments with CK1γ1 knockout cells, demonstrating that inhibition of one isoform would be unlikely to affect the cell fate.

Although CK1γ isoforms show high homology in their primary protein sequences, they are located in different subcellular fractions and have distinct functions in necroptosis. To define the role of CK1γ more precisely, we used the CK1γ-specific inhibitor Gi, which was previously demonstrated to have more selective activity toward CK1γs (CK1γ IC_50_ = 0.029 µM, CK1α IC_50_ = 7.58 µM, CK1δ IC_50_ = 2.62 µM)^26^. CK1γ is known to be palmitoylated at the C-terminus for its membrane localization^36^. Nevertheless, we found that CK1γ3 tends to be located in the more insoluble parts of cells compared to CK1γ1. Unlike RIPK1, RIPK3, and MLKL, which move to insoluble fractions once activated, the subcellular localization of CK1γ remains unchanged during necroptosis induction. Given that the active necrosomes exist in highly insoluble compartments in cells, this might explain why CK1γ3 had the greatest effect on necroptosis among the CK1γ isoforms. Moreover, CK1γ1 and CK1γ3 can form a heterodimer, and CK1γs are found in the insoluble subcellular compartments, raising the possibility that CK1γ1 and CK1γ3 have potential to form oligomers, like MLKL^30^, or higher-order protein complexes as in the RIPK1-RIPK3 amyloid-like structure^28^.

It remains unclear precisely how CK1γ is activated upon necroptosis. Recently, RIPK3 was reported to mediate the production of necroptosis-induced ROS by promoting mitochondrial aerobic respiration via activation of the pyruvate dehydrogenase complex (PDC)^37^. In the case of RIPK1, cysteine residues in the protein were shown to be oxidized by ROS produced during necroptosis, which form disulfide bonds that result in the formation of a high-molecular-weight RIPK1 complex. This oxidization may be important for the autophosphorylation of RIPK1 on Ser^161^, and thus for its activation^38^. Likewise, we observed that overexpressed CK1γ was autophosphorylated, as previously reported^39^, and kinase-dead mutants of CK1γ1 and CK1γ3 do not have pro-necroptotic activity. In addition, an ROS scavenger prevented the accumulation of CK1γ1 and CK1γ3 in necroptosis. These results led us to speculate that CK1γ activation is also influenced by ROS produced in necroptotic cells. However, further investigations are needed to determine whether the increased levels of CK1γ directly affect its activity.

While we found that CK1γ phosphorylates RIPK3 in vitro, it is also important to uncover whether RIPK3 is phosphorylated by CK1γ in cells. When we checked the activation of RIPK3 by examining the slow-migrating RIPK3, the phosphorylated form or it, on SDS-protein gel, it was hard to see any big differences in the migration of RIP3K between WT and CK1γ knockout HeLa/RIPK3 HA cells. However, we observed that the interaction of the activated RIPK3 with MLKL and the amounts of phosphorylated MLKL, the consequences of RIPK3 phosphorylation and activation, were apparently reduced by CK1γ1/CK1γ3 double knockout. In addition, expression of phospho-defective mutant CK1γ3 at Ser^344/345^ also reduced level of the phosphorylated MLKL. Although we were unable to identify a defective phosphorylation of RIPK3 in CK1γ knockout cells and need more extensive analysis to solve this point, these observations imply that RIPK3 might be phosphorylated by CK1γ in cells.

After CK1γ is activated in parallel with RIPK1, RIPK3, and MLKL, it seems to phosphorylate RIPK3, but not vice versa. A possibility of RIPK1 phosphorylation by CK1γ should be confirmed before making more appropriate conclusion, though. The crucial next question to be resolved is whether the phosphorylation of RIPK3 by CK1γ results in the activation of RIPK3. We observed a reduction in the phosphorylation of MLKL Ser^358^ in Gi-treated or CK1γ knockout cells, and consequentially impairment in the formation of MLKL oligomers or the active necrosome. Because MLKL Ser358 is known to be phosphorylated by active RIPK3 and is observed to increase by CK1γ and RIPK3 in our in vitro assays, we believe that CK1γ may be engaged in RIPK3 activation. The decrease of the phospho-MLKL level was not as significant as the decline in the cell death rate caused by CK1γ inhibition (either by an inhibitor or gene knockout), which would be a consequence of the failure in producing a positive feedback loop in CK1γ-inhibited cells. In that case, the additional mechanisms might account for that CK1γ promotes necroptosis via RIPK3 activation: (i) phosphorylation on MLKL at sites other than previously identified residues such as Thr^357^ and Ser^358^ in human MLKL, (ii) translocation of MLKL to the plasma membrane, or (iii) other factors activated by MLKL in the cell membrane. Some MLKL mutants can form oligomers and translocate to the plasma membrane but cannot kill cells^30^. Moreover, the membrane translocation of MLKL alone is not generally considered to be sufficient to cause necroptosis. To date, TRPM7, a cation channel located in the plasma membrane, is the only known mediator that acts downstream of RIPK3/MLKL^40^. Nevertheless, the possibility that CK1γ could help TRPM7, or another as-yet-discovered element, to be activated cannot be ruled out.

In conclusion, it is evident that CK1γs, especially CK1γ1 and CK1γ3, participate in RIPK3-dependent and TNFα-induced necroptosis (Fig. 6c). Stimulated by a necroptotic signal, CK1γs are autophosphorylated, forming a protein complex with the necrosome, and phosphorylate RIPK3 for further activation of necroptosis (Fig. 6c).

## Supplementary information

Supplementary figure legends

Supplementary Figure 1

Supplementary Figure 2

Supplementary Figure 3

Supplementary Figure 4

Supplementary Figure 5

Supplementary Figure 6

Supplementary Figure 7

Supplementary Figure 8

Supplementary Figure 9

Supplementary Figure 10

Supplementary Figure 11

Supplementary Figure 12

Supplementary Table 1

Supplementary Table 2
