# Supplementary material for: Casein kinase-1γ1 and 3 stimulate tumor necrosis factor-induced necroptosis through RIPK3
Source: Cell Death Dis. 2019 Dec 4;10(12):923. doi: 10.1038/s41419-019-2146-4 (PMC6892881; doi:10.1038/s41419-019-2146-4)
Supplement: Supplementary file 1 — Supplementary figure legends [file 41419_2019_2146_MOESM1_ESM.docx]

**Supplementary figure legends**

**Fig. S1. The amounts of CK1γ1 and CK1γ3 proteins, but not CK1γ2 protein, are elevated in necrotic cells.**

HeLa/RIPK3-HA cells were treated with 10 ng/mL TNFα, 100 nM SM-164 and 10 μM IDN-6556 for 4 h (*left*). The levels of CK1γ1, CK1γ2 and CK1γ3 proteins were detected by immunoblotting. Signals on the blots were quantified by densitometry analysis. Bars represent the mean ± SEM from at least three independent experiments. **p*< 0.05 (*right*).

**Fig. S2. The increase of CK1γ1 and CK1γ3 proteins during TSI-induced necroptosis are inhibited by NAC.**

HeLa/RIPK3-HA cells were pretreated for 30 min with 5 mM N-acetyl-L-cystein (NAC) and then treated with 10 ng/mL TNFα, 100 nM SM-164 and 10 μM IDN-6556 in the presence or absence of 10 μM Nec1 or 5 mM NAC.

**Fig. S3. CK1γ inhibitor blocks necroptosis for a longer period.**

HeLa/RIPK3-HA cells were treated 20 ng/mL TNFα, 100 nM SM-164 and 20 μM IDN-6556 for 16 h in the presence or absence of 20 μM Nec1, D4476 or Gi. The cell death rates were determined by counting propidium iodide (PI)-positive cells. Bars represent the mean ± SEM from three independent experiments. **p*< 0.05, ***p*< 0.01*,* ****p*< 0.001.

**Fig. S4. Overexpression of CK1γ1 does not affect apoptosis.**

HeLa cells were left untreated (NT) or treated with 20 ng/mL TNFα and 100 nM SM-164 for 6 h, 100 μM etoposide for 24 h or 8 μg/ml tunicamycin for 48 h. Cell viability was determined by trypan blue exclusion assay (*left*) and the expression of HA-Bcl-2 or CK1γ1 was confirmed by western blotting (*right*). Bars represent the mean ± SEM from at least three independent experiments. **p*< 0.05, ***p*< 0.01, ****p*< 0.001, n.s.; not significant.

**Fig. S5. Downregulation of CK1γ does not affect TS-induced apoptosis.**

(a) HeLa cells or (b) HeLa/RIPK3-HA cells were treated with 20 ng/mL TNFα and 100 nM SM-164 for 7 h in the presence or absence of 20 μM IDN-6556, Nec1, D4476 or Gi. Cell viability was determined by trypan blue exclusion assay (a, *upper*, b) and the cleavage of CK1γ1, RIPK1 and PARP1 was confirmed by western blotting (a, *lower*). Bars represent the mean ± SEM from three independent experiments. **p*< 0.05, ***p*< 0.01*,* ****p*< 0.001, n.s.; not significant.

**Fig. S6. Overexpression of FLAG-tagged CK1γ1 exerts its pronecroptotic activity only in CK1γ1 knockout cells.**

HeLa/RIPK3-HA cells and HeLa/RIPK3-HA/CK1γ1 knockout cells were transfected with either FLAG-CK1γ1 WT or FLAG-CK1γ1 D343A for 24 h, and then treated with 20 ng/mL TNFα, 100 nM SM-164 and 20 μM IDN-6556 for 7 h. Cell viability was determined by CellTiter-Glo (*upper*) and expression levels of endogenous and exogenous CK1γ1 were detected with western blotting (*lower*). Bars represent the mean ± SEM from at least three independent experiments. An unpaired t-test was done in SPSS Statistics. **p*< 0.05.

**Fig. S7. *In vitro* assay showing binding of CK1γ1 or CK1γ3 to MLKL and RIPK3.**

Recombinant His-MLKL, GST-RIPK3 and GST-CK1γ1 or GST-CK1γ3 proteins were incubated overnight at 4 °C as indicated and analyzed by immunoprecipitation (IP) assay using Ni-NTA beads. The immunoprecipitates were analyzed by western blotting using anti-GST and anti-His antibodies

.

**Fig. S8. Gi hardly affects the phosphorylation of RIPK1 or RIPK3.**

(a) HEK 293T cells were transfected with FLAG-RIPK1, FLAG-RIPK3 or FLAG-CK1γ3 for 1 h and treated with 10 μM Gi for 24 h. HeLa cells (b) or HeLa/RIPK3-HA cells (c) were transfected with FLAG-RIPK3 (b) or FLAG-CK1γ3 (c) for 24 h, and then treated with 20 ng/mL TNFα, 100 nM SM-164 and 20 μM IDN-6556 for 3 h. Cells lysates were analyzed by immunoprecipitation (IP) assay with anti-FLAG beads followed by western blotting using phospho-Ser or phospho-Thr antibody. The immunoprecipitates (IP) and whole cell lysates (WCL) were analyzed by western blotting.

**Fig. S9. CK1γ inhibitor Gi does not block MLKL-Q356A-induced cell death.**

HEK 293-Flp-In-T-Rex-hMLKL-Q356A stable cell line was treated with 1 µg/ml tetracycline in the presence or absence of Gi at the indicated concentrations for 24 h and cell survival was determined using CellTiter-Glo.

**Fig. S10. CK1γ inhibitor Gi blocks MLKL oligomerization.**

HeLa/RIPK3-HA cells were treated with 10 ng/mL TNFα, 100 nM SM-164 and 10 μM IDN-6556 for 3 h in the presence or absence of 10 μM Nec1 or Gi. Cell extracts were separated by SDS-PAGE under non-reducing condition and analyzed by western blotting.

**Fig. S11. The ability of RIP3K to bind to MLKL is reduced by CK1γ1 and CK1γ3 knockout.**

HeLa/RIPK3-HA or HeLa/RIPK3-HA/CK1γ knockout cells were treated with 10 ng/mL TNFα, 100 nM SM-164 and 10 μM IDN-6556 for 3.5 h and analyzed by immunoprecipitation (IP) assay using anti-MLKL antibody.

**Fig. S12. Levels of the phosphorylated form of CK1γ3 at Ser^344/345^ in untreated control and TSI-treated cells are almost same.**

HeLa/RIPK3-HA (*left*) or HT-29 (*right*) cells were treated for 3 h with 10 ng/mL TNFα, 100 nM SM-164 and 10 μM IDN-6556 (*left*) or for 5 h with 20 ng/mL TNFα, 100 nM SM-164 and 10 μM IDN-6556 (*right*). Cell extracts were analyzed by western blotting.

**Table S1. The list of cDNAs and putative positive clones**

**Table S2. The number and ratio of phosphorylated peptides at CK1γ3 Ser^344^ or Ser^345^ identified by of liquid chromatography tandem-mass spectrometry (LC-MS/MS).**
