# Supplementary figures and images for: Casein kinase-1γ1 and 3 stimulate tumor necrosis factor-induced necroptosis through RIPK3
Source: Cell Death Dis. 2019 Dec 4;10(12):923. doi: 10.1038/s41419-019-2146-4 (PMC6892881; doi:10.1038/s41419-019-2146-4)

Supplementary figure 1

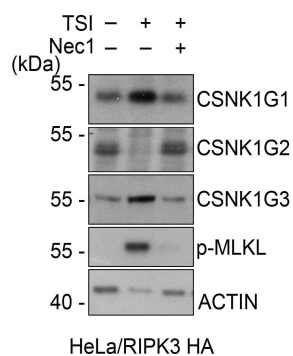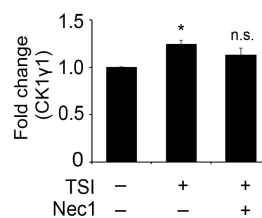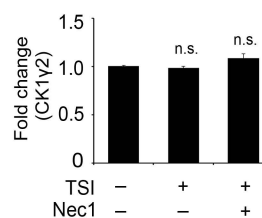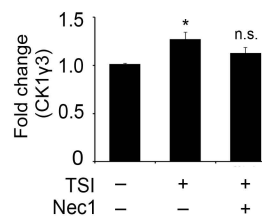

Supplement: Supplementary file 2 — Supplementary Figure 1 [file 41419_2019_2146_MOESM2_ESM.pdf]

## Supplementary figure 2

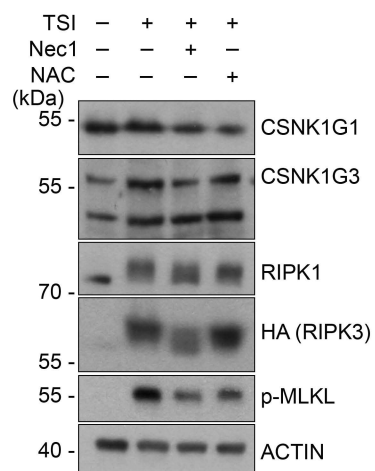

Supplement: Supplementary file 3 — Supplementary Figure 2 [file 41419_2019_2146_MOESM3_ESM.pdf]

Supplementary figure 3

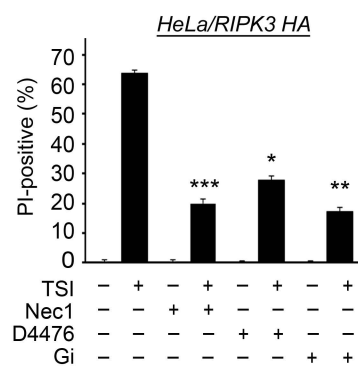

Supplement: Supplementary file 4 — Supplementary Figure 3 [file 41419_2019_2146_MOESM4_ESM.pdf]

Supplementary figure 4

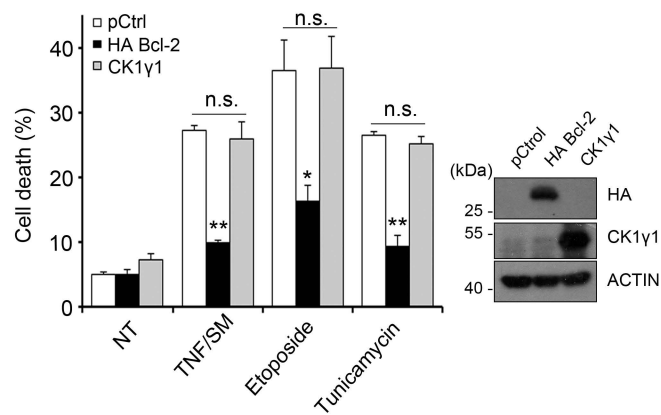

Supplement: Supplementary file 5 — Supplementary Figure 4 [file 41419_2019_2146_MOESM5_ESM.pdf]

Supplemntary figure 5

**a**

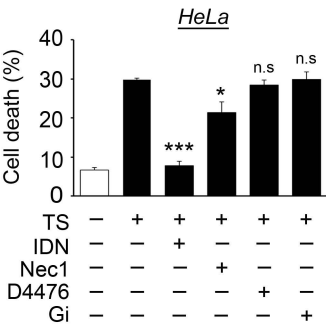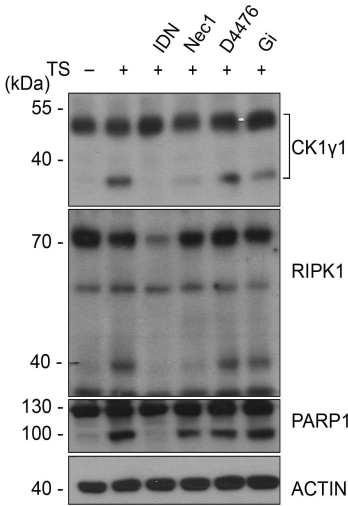

**b**

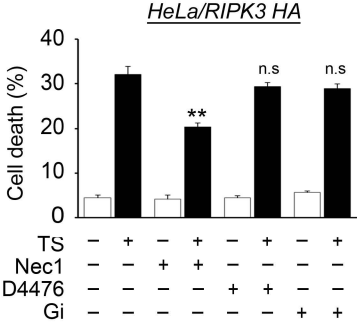

Supplement: Supplementary file 6 — Supplementary Figure 5 [file 41419_2019_2146_MOESM6_ESM.pdf]

Supplemntary figure 6

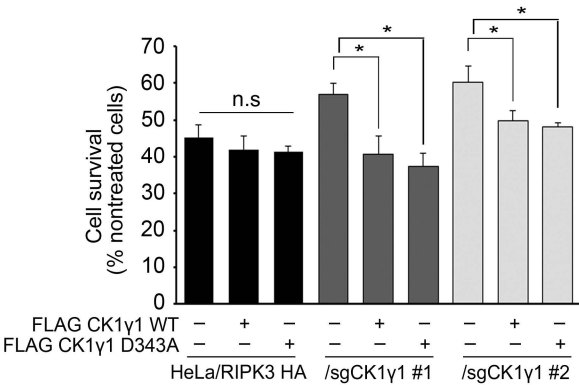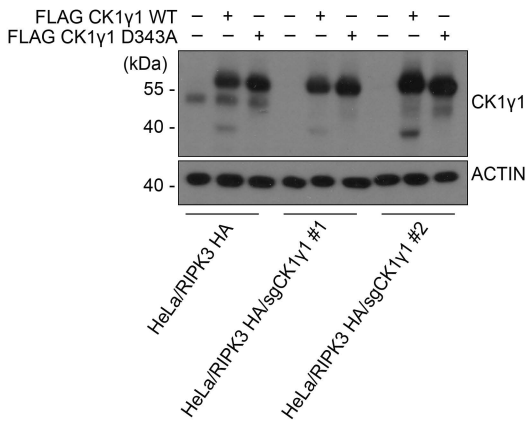

Supplement: Supplementary file 7 — Supplementary Figure 6 [file 41419_2019_2146_MOESM7_ESM.pdf]

Supplemntary figure 7

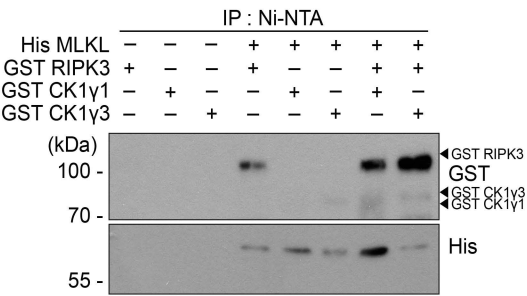

Supplement: Supplementary file 8 — Supplementary Figure 7 [file 41419_2019_2146_MOESM8_ESM.pdf]

Supplemntary figure 8

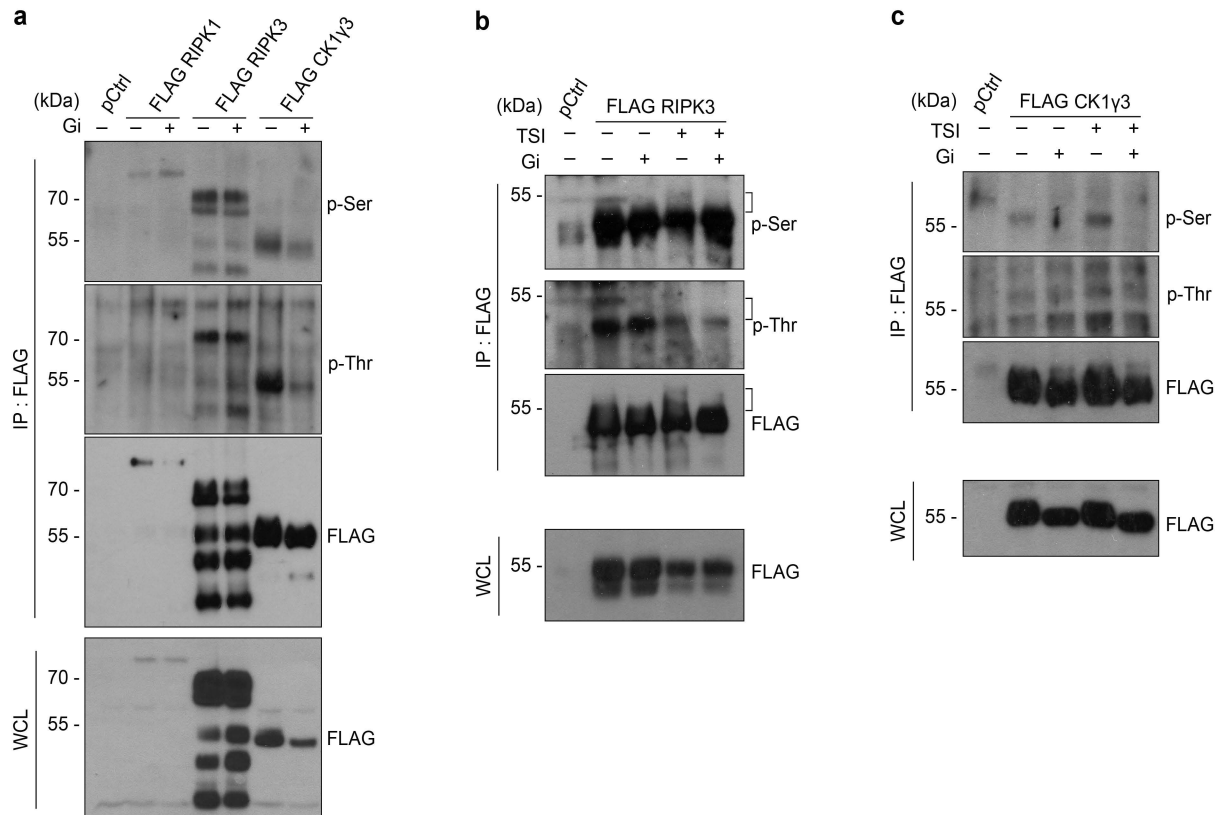

Supplement: Supplementary file 9 — Supplementary Figure 8 [file 41419_2019_2146_MOESM9_ESM.pdf]

Supplemntary figure 9

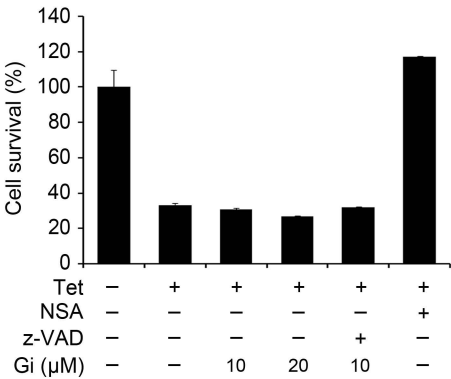

Supplement: Supplementary file 10 — Supplementary Figure 9 [file 41419_2019_2146_MOESM10_ESM.pdf]

Supplemntary figure 10

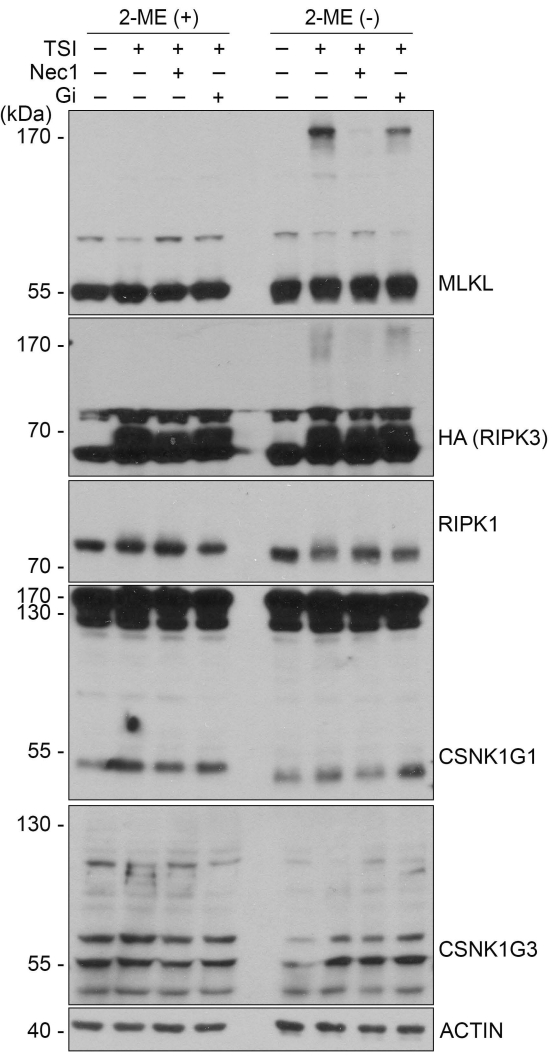

Supplement: Supplementary file 11 — Supplementary Figure 10 [file 41419_2019_2146_MOESM11_ESM.pdf]

Supplemntary figure 11

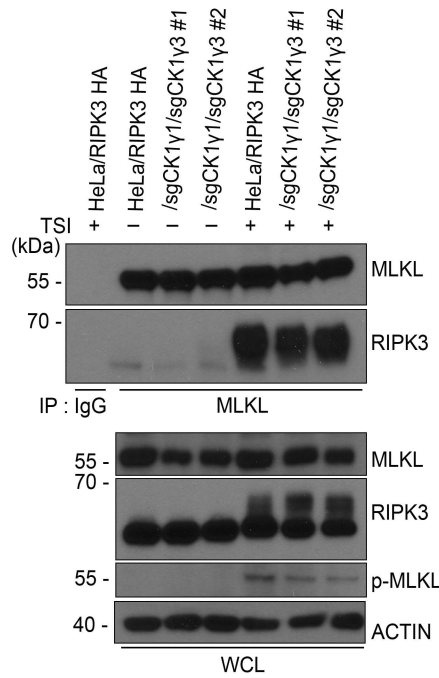

Supplement: Supplementary file 12 — Supplementary Figure 11 [file 41419_2019_2146_MOESM12_ESM.pdf]

Supplemntary figure 12

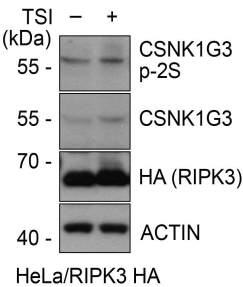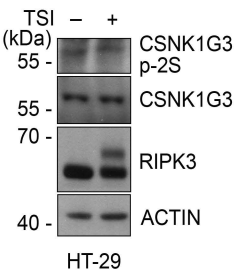

Supplement: Supplementary file 13 — Supplementary Figure 12 [file 41419_2019_2146_MOESM13_ESM.pdf]
