# Supplementary material for: Casein kinase-1γ1 and 3 stimulate tumor necrosis factor-induced necroptosis through RIPK3
Source: Cell Death Dis. 2019 Dec 4;10(12):923. doi: 10.1038/s41419-019-2146-4 (PMC6892881; doi:10.1038/s41419-019-2146-4)
Supplement: Supplementary file 14 — Supplementary Table 1 [file 41419_2019_2146_MOESM14_ESM.pdf]

Table S1. The list of cDNAs and putative positive clones

No.	Kinase	No.		No.		No.	
1	BMPR1A / ALK3	101	BIKE	201	CDK5R2	301	MAP3K8
2	p70S6K1	102	PKCb	202	CKLiK	302	PRKAB1
3	p70S6K1 T389E	103	MKK3	203	IHPK3	303	PRKCSH
4	p70S6K1 T389A	104	ICK	204	FUK	304	ADK
5	BRD3	105	RSKL2	205	MAP2K1	305	NJMU-R1
6	BRSK1	106	HIPK2	206	PCK1	306	MAP3K11
7	Src Y527F	107	ITPK1	207	PFKM	307	PRKRA
8	Pak2	108	CASK(ORF+UTR	208	PIK3R1	308	PCTK1
9	p38	109	GIT2	209	PRKAR1A	309	NAGK
10	Pak3	110	SNARK	210	PRKD2	310	PGK1
11	Pak1	111	SNFILK	211	TBK1	311	CAMKK2
12	CAMK2alpha	112	MST1	212	UMP-CMPK	312	PCTK1
13	PASK	113	TTK	213	FASTK	313	LIMK2
14	MEKK1	114	PAK7	214	IRAK1	314	CCRK
15	MEKK1	115	CINP	215	DYRK3	315	MAPK10
16	MKK7	116	CTK	216	HGS	316	CAMK2B
17	GAA1 / GPAA1	117	STK32A	217	DGKZ	317	CDKL5
18	RPK118 / RPS6KC1	118	Nuak2	218	CKS2	318	CAMK1G
19	PCTAIRE1	119	c-raf	219	PRKCD	319	PHKG1
20	PCTAIRE3	120	PTP4A3	220	FN3K	320	SPHK1
21	PRL3	121	MARK4	221	IKBKG	321	AK3L1
22	hNeK11L(K61R)	122	ALK7	222	AK1	322	PTK2B
23	DOK4	123	AAK1	223	CNK1	323	STK19
24	Myt1	124	CHK2	224	PRKAG1	324	CKMT1
25	Nek2A	125	TSSK1	225	ZAK	325	CDKN1B
26	SBK	126	MEK1	226	MAP3K7IP1	326	RPS6KB1
27	TESK1	127	EphA2	227	BCKDK	327	CDKN2C
28	TESK2	128	PRKCN	228	SNRK	328	CDKN3
29	ULK1	129	ACK1	229	MAP2K3	329	CALM3
30	Tyk2	130	ACTR2	230	NEK2	330	ILK
31	NDR2	131	AKT1	231	PRKCABP	331	CDK5
32	PINK1	132	ALS2CR2	232	PACSIN3	332	SKP1A
33	PINK1	133	BRD2	233	ASK	333	RIPK2
34	PKN2-GFP	134	BUB1	234	PANK4	334	DAPK3
35	ERK8	135	C20orf97	235	MAPKAPK5	335	CKB
36	ERK8(K42R)	136	CABC1	236	CDK2	336	CKB
37	Rac1	137	CAMK2G	237	MAP2K6	337	PRKCSH
38	Cdc42	138	CDK9	238	UCK1	338	CCRK
39	RhoA	139	CHUK	239	IKBKAP	339	CSNK1A1
40	CaMK Ⅰ	140	CSK	240	PLK1	340	GALK2
41	CaMKⅣ	141	CSNK1E	241	RIOK1	341	LYK5
42	CaMKⅡα	142	CSNK1G1	242	NUCKS	342	WSB1
43	CaMK Ⅰ 1-294	143	CSNK2A1	243	TOPK	343	CALM1
44	CaMK Ⅰ 1-317	144	EEF2K	244	CALM1	344	CAMK1G
45	CaMK Ⅰ 1-317	145	ERBB3	245	AKAP10	345	PDPK1
46	CaMK Ⅰ 1-290	146	FES	246	MELK	346	PRKAG1
47	CaMK Ⅰ 1-290	147	FGFR1	247	SPHK2	347	PRKAR1B
48	TAK1	148	FGFR4	248	PTK9	348	CDKN2C
49	TAK1(K63W)	149	FGR	249	PDK2	349	CDK5
50	PDHK1	150	FYN	250	CSNK2B	350	CKMT1
51	PDK1	151	GALK1	251	CDK7	351	NUCKS
52	PDK1 DEAD	152	GPRK2L	252	PMVK	352	PDXK
53	Tlk1(VH113)	153	H11	253	PTK6	353	PRKCH
54	Tlk2(KT46)	154	HCK	254	GUK1	354	NAGK

55 CK I D
56 CK2α
57 Hapsin
58 Hapsin
59 HLPK1
60 TrkC
61 PKC-α
62 PKC-δ
63 PKC-ε
64 PKC-ζ
65 RAGE1
66 RAGE3
67 RAGE4
68 ALK
69 ALK1
70 ALK4
71 AMPK-α1
72 AMPK-α2
73 DIK/RIP4/ANKRD3
74 ALK7
75 ALK5
76 IRE1
77 MLK1
78 MLK2
79 MLK3
80 D/N MLK1
81 D/N MLK2
82 D/N MLK3
83 Erk2
84 GSK3β
85 PKC-α catalytic domai
86 PKC-β catalytic domai
87 PKC-ζ catalytic domai
88 PKC-ε catalytic domai
89 CHK1
90 p38g
91 MKK4
92 MKK7b
93 Akt3
94 JNK1(MARK8)
95 PDHK2
96 PBK
97 TOLLIP
98 YWHAE
99 JNK2d
100 STK36

155 IHPK2
156 IKBKE
157 LCK
158 MAP2K1IP1
159 MAP3K13
160 MAP3K3
161 MAP3K7
162 MAPK13
163 MAPK7
164 MAPK9
165 MAPKAPK2
166 MARK2
167 MATK
168 MLH1
169 MPP1
170 MST4
171 NDUFA10
172 NEK4
173 NEK6
174 NME7
175 NRBP
176 PAK4
177 PDK4
178 PLAU
179 PRKACB
180 PRKCB1
181 PRKCG
182 PRKCL2
183 PRPS1
184 PRPS2
185 RAF1
186 RIOK3
187 RPS6KA5
188 SGKL
189 SSTK
190 STK16
191 STK17B
192 STK3
193 SYK
194 TNK1
195 TRA1
196 TRAP1
197 TRIM28
198 VRK2
199 AURKB
200 CAMK2A

255 DTYMK
256 URKL1
257 SKP2
258 MAPKAPK3
259 STK19
260 PTK9L
261 PLK2
262 AK3
263 CSNK1D
264 STK25
265 MAPKAP1
266 PKMYT1
267 VRK3
268 CALM2
269 MAP3K8
270 PRKCDBP
271 DYRK3
272 PKM2
273 DGKA
274 PRKAA1
275 RPS6KA1
276 ZAK
277 PDPK1
278 PACSIN2
279 PKM2
280 GK
281 RPS6KA4
282 MARK3
283 GUK1
284 SPHK1
285 PANK2
286 SRPK1
287 ZAK
288 HRI
289 DGUOK
290 LIM
291 MAP3K8
292 ADCK2
293 STK25
294 MAP2K5
295 MAP3K8
296 PIK3R2
297 STK11
298 DGUOK
299 CLK3
300 SGK

355 PGK1
356 AKAP9
357 AK2
358 PKMYT1
359 SPHK1
360 CSNK2B
361 AKAP7
362 AK1
363 STK25
364 CDKN1B
365 DOK5
366 CDKN2D
367 CAMK1
368 VRK3
369 GAK
370 PKM2
371 CDK4
372 MAP2K2
373 CDK7
374 SGK
375 LRRK1
376 NTRK3
377 MAP3K11
378 PLK1
379 PCTK3
380 SPHK2
381 TP53RK
382 MVK
383 PDK3
384 PFKL
385 PIP5K1C
386 PTK9L
387 UCK1
388 FLJ11149
389 CSNK1G2
390 MKNK2
391 LIMK2
392 PRKAB1
393 PIK4CA
394 BMX
395 ADCK4
396 AK3L1
397 CARKL
398 PRKAG2
399 AK5
400 ILK

No.	
401	MINK
402	MAP2K6
403	FRK
404	NTRK2
405	MARK3
406	CLK3
407	MAPK11
408	PKLR
409	TEK
410	MAPK14
411	PFKP
412	AK3
413	GK2
414	STK22C
415	CAMKK1
416	PIP5K2B
417	ADCK4
418	ZAP70
419	CDK6
420	PIP5K3
421	GK
422	MAP2K3
423	PHKB
424	MGC45419
425	CAMK1G
426	CAMK2D
427	TLK1
428	MAP3K14
429	STK24
430	ADRBK1
431	STK32B
432	TIE
433	CAMK1D
434	PIK3CG
435	PRKACG
436	FASTK
437	PGK2
438	AXL
439	HK3
440	FLT3LG
441	EKI1
442	K1H9
443	MAST205
444	DRAK1
445	EIF2AK4
446	DDR2
447	SAST
448	KPI2 (LMTK2)
449	KPI2
450	SRM
451	PKCa
452	Trad
453	STK39
454	MAP3K10/MLK2

No.	
501	NEK9
502	TRRAP
503	MAP3K2
504	MAP3K2/MEKK2
505	ACVR2
506	PYK2
507	JNK3(MAPK10)
508	Slob
509	p38a
510	FLJ10074
511	CK1g2
512	PANK3
513	WBP2
514	MST2
515	EphA4
516	SGK3
517	CDK5R1
518	TIE2
519	CKMT2
520	PFKFB4
521	PDHK4
522	MAPK6
523	PIK3R3
524	PDHK1
525	EphA7
526	TSSK3
527	MAP4K2/GCK
528	NIK
529	JNK2 (MAPK9)
530	PDK1
531	JAK3
532	MLKL
533	GSK3A
534	MAST1
535	ALK2
536	PAK6
537	TESK1
538	BARK1
539	ADRBK2
540	PRKCL1
541	HSA250839
542	TIE1
543	EphA8
544	FLT1
545	p70S6K
546	PDHK3
547	
548	
549	
550	

No.	Phosphatase
1	CDKN3
2	DUSP6
3	PP
4	PPP1R7
5	PPP1R1B
6	PPP1R8
7	ENPP6
8	PPP3CC
9	THTPA
10	PPP2R1A
11	ACP1
12	PPP5C
13	PSTPIP1
14	PR48
15	PPA2
16	PSTPIP2
17	PTPN1
18	PPP2R2D
19	PTPN4
20	PPP2R5D
21	PPP1R12C
22	PTPN9
23	FLJ32332
24	PR48
25	INPP1
26	ACYP2
27	DUSP22
28	PPM1D
29	RNGTT
30	PPM1F
31	PPP2CB
32	PFKFB4
33	ALPP
34	ACP6
35	PPM1A
36	PTPNS1
37	PTPRA
38	FBP1
39	PPM1B
40	PHPT1
41	PPP2R2B
42	PPP3R1
43	PPP3CA
44	DUSP10
45	PTPNS1L2
46	PHACTR4
47	PPP1R12B
48	PPP2CA
49	PSTPIP2
50	PPP3CB
51	ACYP1
52	PNKP
53	MINPP1
54	DUSP3

No.	
101	DUSP12
102	MKPX
103	PTPN7
104	branching-enzyme interacting DUSP
105	serine/threonine/tyrosine interacting-like 1
106	PTPRO
107	PTP non-receptor type 18
108	PTPase-alpha
109	PTPRM
110	PTPRB
111	PTPRH
112	IAR/receptor-like PTP
113	PTP non-receptor type 4
114	PTPRO
115	hematopoietic cell phosphatase
116	PTPRT
117	PTPRS
118	PTPN2 (1-1256)
119	PTPRZ1
120	PTPN13
121	PTPN3 (1233-1916) WT =2742(full)
122	PTPRE (1-2387) full length
123	PTPRK
124	PTPN14
125	CDC25C
126	ACP1
127	sphoserine/threonine/ty rosine interactionprotein

455 PRKAG2/AMPK
456 MRCKb
457 STLK5
458 IKKb
459 EphB4
460 JNK1 (MAPK8)
461 NEK4
462 ULK1
463 ERBB2
464 NEK2
465 AKT2
466 PTK2
467 CDC7
468 PKACa
469 RIOK2
470 PKN3
471 DYRK4
472 EVA1
473 PLK2
474 MYT1
475 PLK4
476 ADCK5
477 HER3/ErbB3
478 PAK1
479 MAST4
480 LATS1
481 MAST2
482 CSNK1G3
483 MNK1
484 PRKX
485 RSK1
486 RYK
487 Haspin
488 PLAUR
489 IHPK1
490 CHK2
491 NRBP1
492 MSK2
493 MASTL
494 ADCK3(CABC1)
495 GADD45G
496 TGFbR1
497 CK2a2
498 MAP3K4
499 LKB1 (STK11)
500 p70S6Kb

55 PPP2CB
56 PPP6C
57 DUSP14
58 PTPNS1
59 PTEN
60 PTPN9
61 DUSP6
62 PPP3CB
63 PTN
64 PTPN12
65 DUSP11
66 MTMR9
67 PTPRR
68 EPM2A
69 PPP2R1A
70 PPP2R2A
71 FLJ20442
72 MK-STYX
73 DUSP5
74 FLJ20300
75 PPM1A
76 MGC1136
77 PPP1CB
78 DUSP1
79 SET
80 PTPN11
81 PTPN2
82 hypothetical protin / RIKEN
83 myotubularin related protein 8
84 DUSP23
85 hSSH-1L
86 PTPRO
87 DUSP15
88 KIAA1075 protein
89 KIAA protein
90 DUSP13
91 tensin
92 MMAC1
93 PTP4A2
94 KAP1
95 DUSP1
96 DUSP2
97 DUSP3
98 DUSP6
99 DSUP7
100 DUSP9
