# Supplementary material for: Casein kinase-1γ1 and 3 stimulate tumor necrosis factor-induced necroptosis through RIPK3
Source: Cell Death Dis. 2019 Dec 4;10(12):923. doi: 10.1038/s41419-019-2146-4 (PMC6892881; doi:10.1038/s41419-019-2146-4)
Supplement: Supplementary file 15 — Supplementary Table 2 [file 41419_2019_2146_MOESM15_ESM.pdf]

**Table S2. The number and ratio of phosphorylated peptides at CK1γ3 Ser344 or Ser345 identified by of liquid chromatography tandem-mass spectrometry (LC-MS/MS).**

| | | The number of peptides identified phosphorylated:non-phosphorylated (ratio, %) | |
|---|---|---|---|
| **Phospho-peptide** | **Site** | **NT** | **TSI** |
| QLPTPVGAVQQDPALS*SNREAHQHR | S344 | 15:78 (16.13 %) | 22:87 (20.18 %) |
| QLPTPVGAVQQDPALSS*NREAHQHR | S345 | 9:84 (9.68 %) | 15:94 (13.76 %) |
